# An updated survey of freshwater fishes within Letchworth State Park and surrounding area's of the Genesee River

**DOI:** 10.1002/ece3.11333

**Published:** 2024-04-30

**Authors:** G. Gascoigne, J. Assad, D. Patterson, J. Brillhart, V. DiLeo, R. Williams

**Affiliations:** ^1^ The College of Wooster Wooster Ohio USA; ^2^ Fordham University New York City New York USA

**Keywords:** biodiversity, conservation, fish survey, Genesee River, Letchworth State Park

## Abstract

The goal of this study was to gather information about freshwater fishes in Letchworth State Park (42.615275° N, −77.992825° W), a portion of New York State‐owned land located in the Genesee River Watershed that lacks known data about its fish diversity. Fish collection took place between 2017 and 2019 in the Genesse River upstream and downstream of the falls using electrofishing, gill, hoop, and seine netting. This was the first attempt at a comprehensive survey of this portion of the river, which allowed for a baseline to be established regarding fish biodiversity in the region. The updated total number of species found in this portion of the Genesee River was 25, 22 of which were newly identified downstream of the falls in Letchworth State Park. We encourage further collection and continuation of this survey with consistent sampling techniques to raise awareness about the importance of freshwater fish diversity in stream ecosystems across the globe.

## Graphical abstract

We gathered fish species survey data from a region of the Genesee River in New York that has been insufficiently accessed to provide a baseline number of fish species in Letchworth State Park. We believe our fish species list can be used to identify trends in diversity in future studies and that our study will serve as preliminary data for the continuation of this work. We identified a total of 25 species of fish in this study, 22 of which were first collected within Letchworth State Park, below the falls.

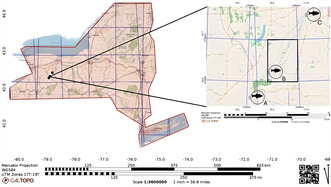

**Objectives**

- Gather fish species survey data from a part of the Genesee River in Letchworth State Park that will serve as preliminary data for future studies.
- Advocate for habitat protection, government or private funding, and the development of recovery and management programs in a popular public state park sponsored by NYSDEC.
- Educate the public on the ecological and economic importance of biodiversity and of freshwater ecosystems, especially the Genesee River.

## 1 INTRODUCTION

### 1.1 Freshwater biodiversity

Freshwater ecosystems are considered a source of consumable water that can be used for agricultural, commercial, and domestic purposes (Gleick, 2003). In terms of biological value, freshwater supplies some of the richest ecosystems on the planet. Though only 0.01% of freshwater can be found on the earth's surface, it supports a plethora of life, specifically 40% of global fish diversity (Dudgeon et al., 2006; McAllister et al., 1997). It is imperative, therefore, to protect the biodiversity of freshwater ecosystems, given the dependence life has on services provided by rivers, lakes, streams, and wetlands (Apostolaki et al., 2020).

This goal can be achieved through long‐term monitoring, as it is a valuable tool in the evaluation of species, species richness, and the quantification of diversity through the Simpson and Shannon indices. The Simpson index calculates the probability that any two individuals randomly selected from a specified habitat will belong to the same species. The Shannon diversity index quantifies diversity in a population by comparing the population's richness (number of species in a habitat) and evenness (number of unique species; Hill, 1973; Hurlbert, 1971). Both of those indices were employed in a study that found an increase in biodiversity in the Pinang River in Malaysia over the course of their study, despite land conversion to urban, agricultural, and recreational usage (Yunus et al., 2004). Another study utilized the Shannon index to quantify vegetation biodiversity and found that the increase in cultivated and residential areas has led to a decrease in vegetation and water yield in China's Min River (Ma et al., 2020). The interpretation of this data should lead to the formulation of specific policies to prevent further debilitation of natural habitats (Wooldridge et al., 1999). For those policies to effectively take place, community involvement is necessary and can be achieved by sharing information through public presentations. In this study, we propose an updated species list that will provide valuable data for biodiversity monitoring regarding the freshwater ecosystem within Letchworth State Park.

### 1.2 Letchworth State Park and the Genesee River: Review of previous fish surveys

This project's aim was to survey the Letchworth State Park portion of the Genesee River above and below the falls and establish a reliable baseline of fish species composition that seemed missing or unspecified from previous studies. Fish surveying is a technique frequently used in the United States to develop management plans or recovery programs, identify habitats, and assess fish distribution and abundance (Carlson et al., 2016; Carlson & Daniels, 2004). Prior to this study, Carlson & Daniels (2004) identified data needs in all 19 watersheds in New York and attempted to quantify fish assemblages in each of them, including the Genesee, but information on the fish species in Letchworth State Park was limited. It is unclear whether Carlson and Daniels collected data within the park, but they did comment on the two natural barriers that are impassable to fish: the Letchworth and Rochester waterfalls. Because of these barriers, the species composition of fishes varies for different sections of the river upstream and downstream of the waterfalls (Carlson & Daniels, 2004).

Five sections of the Genesee River, including Letchworth State Park, were regularly surveyed for trout and Smallmouth bass in 2010, 2014, 2017, and 2020, between March and October, by an Angler Diary Program, sponsored by the New York State Department of Environmental Conservation (DEC) Region 9. Unlike Carlson and Daniel's (2004) comprehensive survey of fishes in the river and watershed, the Angler Diary program's aim was focused on the two species of interest, with only 1 bass recorded in 2020 (New York Department of Environmental Conservation [NYSDEC], 2018, 2020). This species overlapped with another smallmouth bass recorded 0.2–0.5 miles below the lower falls of the park in 1959 (Carlson et al., 2016). Furthermore, 7 species of fish were noted between the falls but were not listed in *A Biological Survey of the Genesee River System* (1927). The metadata file that accompanied the Atlas specified 6 of these 7 fish mentioned between the upper and lower falls (Carlson et al., 2016). Therefore, no consistent sampling was conducted past Portageville or within the park boundaries, and no consistent monitoring has been published by these initiatives (NYSDEC, 2018, 2020).

Based on the review of species data from previous surveys, we targeted the gap in fish species diversity in Letchworth State Park as the goal of this study. We used an atlas published in 2016 that included data from fishes recorded over three survey periods in the Genesee Watershed, including Carlson & Daniel's published 2004 survey, to interpret which fishes caught in our study were novel to our sampling site in the park. Review of the atlas identified the presence of 104 fish species in the Genesee Watershed and 89 fish species in the Genesee River (Appendix 1; Carlson et al., 2016). We then hypothesized that more species would be sampled in the survey we conducted at Letchworth State Park than had previously been documented.

## 2 MATERIALS AND METHODS

### 2.1 Study system

Letchworth State Park, founded in 1907, is an area of 14,500 acres in Upstate New York that encompasses 30 km of the Genesee River and surrounding lands, stretching from Portageville (42.5686° N, 78.0411° W) to Mt. Morris (42.7256° N, 77.8742° W), in regions 8 and 9 as designated by the New York State Department of Environmental Conservation (NYSDEC, n.d.). It is known as the “Grand Canyon of the East,” with heights over 167 m and including three waterfalls (Cook, 2011; Ensminger et al., 2016; Spinola et al., 1998; https://dec.ny.gov/things‐to‐do/watchable‐wildlife/sites/letchworth‐state‐park; https://dec.ny.gov/about). Letchworth is in the Genesee River Watershed, which falls in Pennsylvania and New York states. A majority of the drain 6146 km^2^ of the drainage area is in New York State. The Genesee River is almost entirely within New York State, except for the first 24 km in Pennsylvania. The river is the main water source for the drainage area that flows north for about 225 km before empyting into Lake Ontario (New York State Department of Environmental Conservation, n.d.). The park was named after the industrialist William Letchworth, who noticed the negative impacts logging was having in the region in the beginning of the nineteenth century and purchased the land in order to better preserve the natural landscape (Bow, 2013). Much of the infrastructure and many of the facilities present in the park today are due to the Civilian Conservation Corps program, created by the Emergency Conservation Act, signed by the former president Franklin Roosevelt in 1933, aiming to develop conservation projects toward Letchworth State Park (Cook, 2015).

### 2.2 Study design

**FIGURE 1:**
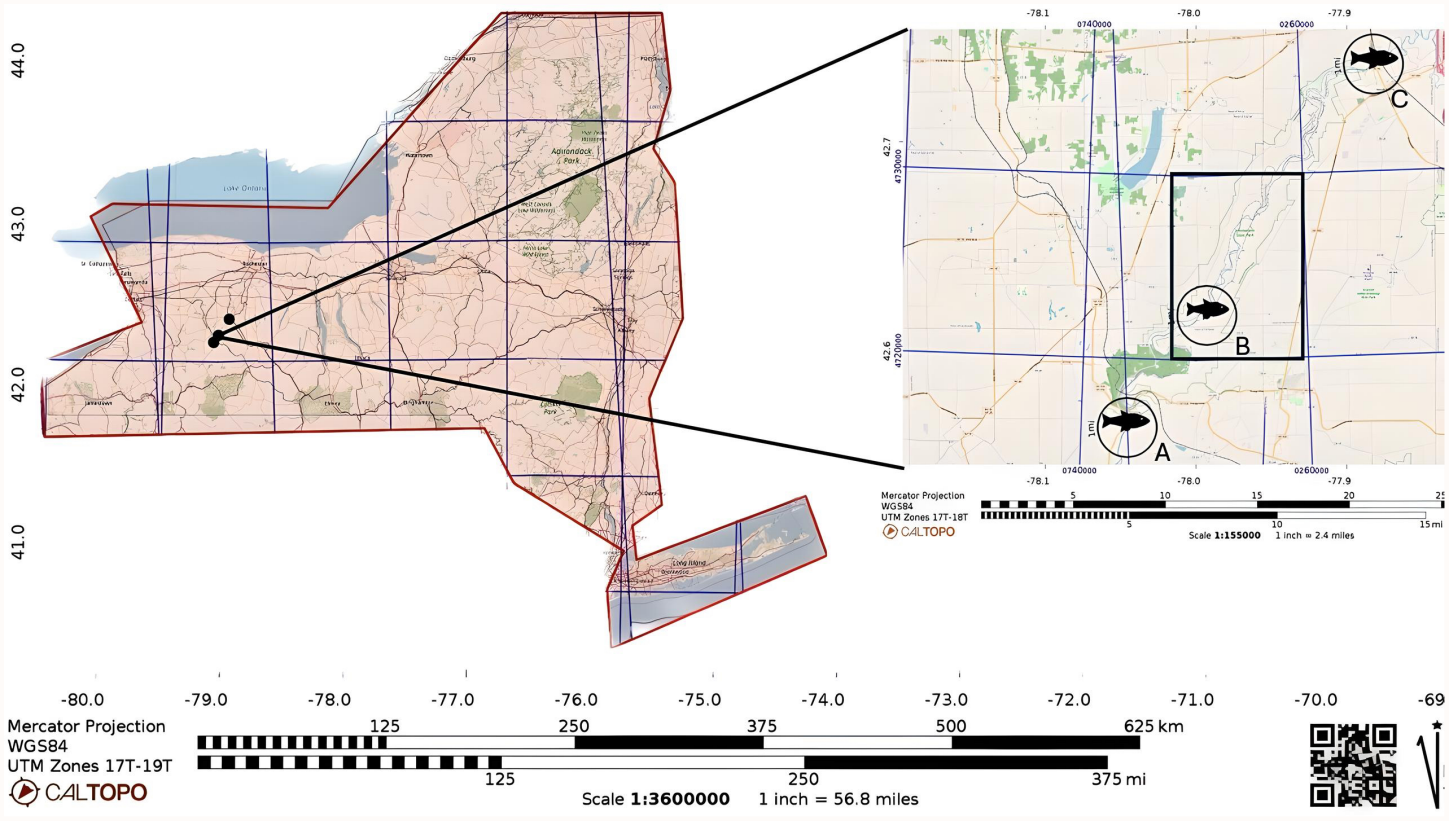
Field study sites. The map on the left side represents the state of New York. The inset map (right side) represents the portion of the Genesse river that was sampled in Letchworth State Park and surrounding area's of the Genesee River. The fish icons represent where data were collected, at site A (42.571026° N, −78.040590° W) near Portageville, B (42.615275° N, −77.992825° W) inside park, and C (42.738133° N, −77.882228° W) near Mt. Morris. The black box inside the inset map represents Letchworth State Park. The maps also have a north arrow, a scale, and latitudinal and longitudinal coordinates which are indicated by the degree measurements and blue lines.

Fishes were collected between August and November over 3 years (2017–2019) in three different locations labeled A, B, and C (Table 1, Figure 1). Site A was located upstream of the park in Portageville (42.571026° N, −78.040590° W) and sampling took place on either side of the bridge on County Rd. 436, which is the border of Sections 4 and 5 (sections described in the *Review of DEC region 9 angler diaries*). Site B was located within the park itself, downstream of the falls, and site C was downstream of the Mt. Morris dam, past Section 5 (42.738133° N, −77.882228° W). Permission to collect fishes throughout this study was granted by the New York State Department of Environmental Conservation (License to Collect or Possess: #2029).

**TABLE 1:** Methods of capture during fish survey.

Year of sampling	Sites sampled	Method of collection	Number of fish species collected
2017	B	Hoop netting	8
2018	A*, B, C*	Hoop netting*, seine netting, gill netting, electrofishing	20
2019	A, B	Hoop netting	7

*Note*: List of the years sampling took place (1st column), which sites were sampled each year (2nd column), the techniques used (1st column), and the number of fishes collected yearly (4th column). *Asterisk denotes only hoop net capture technique took place at that site in 2018.

### 2.3 Field methods

Various methods were utilized to collect fishes across sites, including hoop, gill, and seine netting, and electrofishing (Table 1). Hoop netting is an entrapment technique that requires manual set up at the bottom of a creek or river and is then collected the next day (Hubert et al., 2012). Hoop and gill nets were chosen to capture larger fish, while seine and electrofishing were used to capture smaller fish. Electrofishing is the use of electric fields in water to shock fishes and then collect them (Snyder, 2003). The use of each method depended on the accessibility of the site and access to boats and equipment.

In 2017, only site B was sampled, and the method chosen was hoop netting, since its set up was possible given site B's accessibility by foot and it was the only equipment available through the institute. In 2018, electroshocking and gill and seine netting were made possible by NYSDEC. Access to challenging locations inside the park upstream of site B was possible via white‐water rafting trips sponsored by Adventure Calls, NY. Unfortunately, fishes were not caught during rafting excursions, but sampling success continued during that field season using hoop nets via foot access at sites A and C. In 2019, only hoop nets were used, and fishes were captured from sites A and B only. The way fishes were collected aimed to reduce sampling bias as nets were dropped overnight and we were unaware of which fishes would be collected the next morning.

Between 1 and 3 hoop nets were left out overnight during each field trip before collection the next day. Once collected, fishes were laid flat and photographed for data collection. The images were later reviewed to identify individual fish species by at least two investigators from NY and OH for all years. Identification was completed based on a set of distinctive biological features and exterior markings for each (Fish ID, n.d.; Kraft et al., 2006). In 2018, fish species captured during electrofishing and gill and seine netting at site B were identified during collection by the NYSDEC (Table 2).

**TABLE 2:** Fish collected in Letchworth State Park.

Fish species	Scientific name	2017	2018	2019	Field site	Novel to site B (Carlson et al., 2016)
Rock bass	*Ambloplites rupestris*	X		X*	B, A* (*caught within section 5)	Y
Yellow bullhead	*Ameiurus natalis*	X			B	Y
Central stone roller	*Campostoma anomalum*		X		B	
Spotfin shiner	*Cyprinella spiloptera*		X		B	Y
Greenside darter	*Etheostoma blennioides*		X		B	Y
Rainbow darter	*Etheostoma caeruleum*		X		B	Y
Fan‐tailed darter	*Etheostoma flabellare*		X		B	Y
Cutlip minnow	*Exoglossum maxillingua*		X		B	Y
Northern hogsucker	*Hypentelium nigricans*	X	X		B	Y
Pumpkinseed sunfish	*Lepomis gibbosus*		X	X	B	Y
Bluegill	*Lepomis macrochirus*	X	X	X	B	Y
Smallmouth bass	*Micropterus dolomieu*		X	X	B	
Largemouth bass	*Micropterus salmoides*	X	X	X	B	Y
White perch	*Morone americana*		X		B	Y
Black redhorse	*Moxostoma duquesnei*			X	B	Y
Golden redhorse	*Moxostoma erythrurum*		X		B	Y
Shorthead redhorse	*Moxostoma macrolepidotum*	X	X	X	B, C	Y
Greater redhorse	*Moxostoma valenciennesi*		X		C	
Stone cat	*Noturus flavus*		X		B	Y
Yellow perch	*Perca flavescens*		X		B, C	Y
Logperch	*Percina caprodes*		X		B	Y
Bluntnose minnow	*Pimephales notatus*		X		B	
White crappie	*Pomoxis annularis*	X			B	Y
Black crappie	*Pomoxis nigromaculatus*	X			B	Y
Longnose dace	*Rhinichthys cataractae*		X		B	Y

*Note*: Common name, scientific name, year, location, and source of fish identification for fish collected between 2017 and 2019. The symbol “Y” indicates whether fish were identified during the most recent survey of the Genesee watershed between 1988 and 2015 at site B (Carlson et al., 2016). Symbol “X” denotes the identification of species at field sites “A,” “B,” and “C” (details in Figure 1). Corresponding “X” and letter “C” underline indicates that fish were caught from that field site that year.

### 2.4 Review of DEC region 9 angler diaries

Reports from 2010, 2014, 2017, and 2020 Angler Diarists were retrieved from the NYSDEC Region 9 office and smallmouth bass and trout data sampling was reviewed to determine where catch overlapped with our collection sites A and B (Cornett & Stratton, 2021; Cornett & Zanett, 2018; NYSDEC, 2018, 2020).

Anglers focused on data collection of all trout (Rainbow trout, Brown trout, and Brook trout) and Smallmouth bass throughout the Genesee River, dividing it into five sections: Section 1 from Pennsylvania to Wellsville, Section 2 catch and release, Section 3 from Wellsville to Route 86 below Belmont, Section 4 from Route 86 to Portageville, and Section 5 from Portageville to the Livingston County line in Letchworth State Park.

### 2.5 Public education

In order to educate the public on the importance of freshwater biodiversity in the Genesse River, research cohorts presented findings at the Humphrey Nature Center in Letchworth State Park in 2017 and 2019. Similar campus presentations were made annually at Houghton University during the 3 years of our project. We hope that these initiatives served as a step toward the conservation and protection of such a valuable ecosystem.

## 3 RESULTS

Each fall, between August and November of 2017, 2018, and 2019, fishes were captured in Letchworth State Park in New York State and surrounding areas with the goal of recording which fishes occupy that area of the Genesee River. Altogether, a total of 25 species were identified (Tables 1 and 2). All the collected species were previously found within the Genesee watershed (Carlson et al., 2016). Of these 25 species, 24 were found at site B. There was one fish species, the Greater redhorse, that was found at site C but was not caught at site B (Table 2).

### 3.1 Data in context

The last attempt to inventory all species in the Genesee Watershed analyzed three different survey periods and identified 104 fish species, 89 of which are in the Genesee River. Of the fishes present in the watershed, 77 were located above the falls and 98 were below the falls. Comparing those previous surveys with the present study, we concluded that all the species from our survey have been collected in the Genesee Watershed and River before, and 2 of them have been previously sampled at site B in Letchworth State Park. Moreover, one of the species collected in Letchworth State Park, Rainbow darter (*Etheostoma caeruleum*), was also identified in the Genesse Watershed as newly introduced (Carlson et al., 2016).

A review of fish surveyed at sites A, B, and C from Carlson and Daniels 2016 Atlas were included to recognize overlap in fish species already identified at site B (Carlson et al., 2016).

Of the 104 Genesee Watershed fish species, two were collected near site B after 1977 (Bluntnose minnow, Central stoneroller). Other fish have been inconsistently observed at sites A and C, upstream and downstream of the falls, respectively: Cutlip minnow, Black redhorse (A, pre 1977); Greater redhorse, White perch, Stonecat, Shorthead redhorse, Bluegill, Rainbow darter, and Black crappie (C, post 1977); Pumpkinseed and Log perch (A pre 1977, C post 1977); Fantail darter, Golden redhorse, Smallmouth bass, Spotfin shiner (A, C post 1977); Yellow perch (A post 1977); Northern hogsucker, Largemouth bass (A post 1977; C pre 1977) and Rock bass, Greenside darter, and Longnose dace have only been sampled before 1977 at both sites (Carlson et al., 2016).

### 3.2 Public outreach

To increase protection strategies toward freshwater ecosystems, we tried to motivate the public by sharing information about the local fish biodiversity. In 2017, our presentation at Letchworth State Park was advertised in the Wellsville Daily Reporter, leading to a connection with a local rafting company, which increased accessibility to unreachable areas (Staff reports, 2017). We then expect increased community involvement in protecting the park, given public awareness about biodiversity.

## 4 FUTURE DIRECTIONS FOR LETCHWORTH STATE PARK

This novel biodiversity analysis was motivated by the inconsistency in the species monitored in preceding studies, along with the lack of precision regarding fish locations. The distinct nature of the present study is its pioneer role in recording fish species in the freshwater ecosystem within Letchworth State Park, a geographically unique area whose fish biodiversity has not yet been quantified. The data collected at Letchworth State Park allowed us to establish a baseline for fish biodiversity in the area, and we concluded that the increase in the number of sites sampled and methods of capture influences the maximum number of fish species caught.

Thus, we highlight the importance of our survey to ongoing assessments of freshwater biodiversity in stream ecosystems, not only in this area but globally. This preliminary study can, therefore, serve as a baseline for long‐term monitoring in future studies. We found that between our survey, review of the 2016 Atlas, and NYSDEC's diary program; 22 of the 24 species identified at Letchworth State Park's site B were first captured in this study (Table 2).

The greatest number of fishes identified in 2018 does not imply an actual increase in fish species that year, but it was the only year when the most effective method of sampling was utilized, and all three field sites (A, B, C) were visited. Electrofishing in 2018 led to the capture of most species of fishes because this technique's electrical current guides and immobilizes a greater number of fishes toward the directionality of the net (Dodds & Whiles, 2020). It is imperative to acknowledge that the change in the number of fish species over the years is due to divergent collection methods and variation in the sampling sites given weather restrictions. Thus, annual changes in fish species number are not indicative of an actual biodiversity change. Moreover, each capture technique specialized in catching fish of distinct sizes. Electrofishing and seine netting methods capture smaller fishes, while gill nets and hoop nets capture larger fishes (Hubert et al., 2012; Lapointe et al., 2006). Since each method was not used at every site or every year, maximum collection potential may not have been reached, and statistical analysis regarding population changes and biodiversity indices was not performed. However, our sites could be used in the future to measure physical and behavioral changes in fishes from the two populations separated by the Letchworth environmental barrier (upper and lower falls) by tracking marked individuals over time (Rodríguez, 2002). In order for this type of study to take place, consistent sampling sites and fishing techniques are necessary over time to assure repeatability, and statistical significance of future studies (Guillard and Verges, 2007). This way, population changes, species richness, and invasive species throughout the park can be more accurately analyzed (Lee Ii et al., 2008).

In the future, efforts should be made to continue monitoring fish biodiversity in the park. We recommend, therefore, sampling be conducted over a greater period of time, using the same methods at each site annually, so that the recommended Shannon and the Simpson indices could be applied. Ongoing consistent fish sampling could lead to a more robust and representative understanding of fish diversity in the park (Diversity Indices, 2024). Outreach efforts should also be stimulated as they led to increased networking, awareness, and subsequent sampling efforts. Pursuing these community partnerships would improve the data we could eventually collect if those relationships continued. Continued research in this field may also assist in advocacy for habitat protection, government or private funding, and the development of recovery and management programs in the popular public state park.

## AUTHOR CONTRIBUTIONS

**R. Williams:** Conceptualization (lead); data curation (equal); formal analysis (lead); funding acquisition (lead); investigation (lead); methodology (lead); project administration (lead); supervision (lead); validation (equal); visualization (equal); writing – original draft (equal); writing – review and editing (equal). **D. Patterson:** Data curation (equal); formal analysis (equal); investigation (equal); validation (equal); visualization (equal); writing – original draft (equal); writing – review and editing (equal). **G. Gascoigne:** Formal analysis (equal); investigation (equal); validation (equal); visualization (equal); writing – original draft (equal); writing – review and editing (equal). **N. J. Assad:** Data curation (equal); formal analysis (equal); investigation (equal); validation (equal); visualization (equal); writing – original draft (equal); writing – review and editing (equal). **J. Brillhart:** Investigation (equal); validation (equal); visualization (equal); writing – review and editing (equal). **V. DiLeo:** Formal analysis (equal); validation (equal); visualization (equal); writing – original draft (equal); writing – review and editing (equal).

## FUNDING INFORMATION

This study did not receive any specific grants from funding agencies in the public, commercial, or not‐for‐profit sectors. Funding for this project was made possible through Houghton University, NY and the College of Wooster, Ohio.

## PERMISSION

We received permission to complete this study from the New York Department of Environmental Conservation and the New York State Office of Parks, Recreation and Historic Preservation.

## Data Availability

The data that support the findings of this study are openly available in [DRYAD] at DOI: 10.5061/dryad.jdfn2z3gq (Williams et al., 2024).

## APPENDIX 1

### Atlas of Inland Fishes of New York

**Table:**

Fish species	Scientific names	Genesee watershed	Genesee River
Alewife	*Alosa pseudoharebgus*	X	X
Allegheny pearl dace	*Margariscus margarita*	X	X
American brook lamprey	*Lethenteron appendix*	X	X
American eel	*Anguilla rostrata*	X	
Atlantic salmon	*Salmo salar*	X	X
Banded darter	*Etheostoma zonale*	X	X
Banded killifish	*Fundulus diaphanus*	X	X
Bigmouth shiner	*Notropis dorsalis*	X	X
Black bullhead	*Ameiurus melas*	X	X
Black crappie	*Pomoxis nigromaculatus*	X	X
Black redhorse	*Moxostoma duquesni*	X	X
Blackchin shiner	*Notropis heterodo*	X	X
Blacknose shiner	*Notropis heterolepis*	X	X
Blackside darter	*Percina maculata*	X	X
Bluegill	*Lepomis macrochirus*	X	X
Bluntnose minnow	*Pimephales notatus*	X	X
Bowfin	*Amia calva*	X	X
Brassy minnow	*Hybognathus hankinsoni*	X	
Bridle shiner	*Notropis bifrenatus*	X	
Brindled madtom	*Noturus miurus*	X	X
Brook silverside	*Labidesthes sicculus*	X	X
Brook stickleback	*Culaea inconsans*	X	X
Brook trout	*Salvelinus fontinalis*	X	X
Brown bullhead	*Ameiurus nebulosus*	X	X
Brown trout	*Salmo trutta*	X	X
Central mudminnow	*Umbra limi*	X	X
Central stoneroller	*Campostoma anomalum*	X	X
Chain pickerel	*Esox niger*	X	X
Channel catfish	*Ictalurus punctatas*	X	X
Chinook salmon	*Oncorhynchus tshawytscha*	X	X
Cisco	*Coregonus artedi*	X	
Coho salmon	*Oncorhynchus kisutch*	X	X
Common carp	*Cyprinus carpio*	X	X
Common shiner	*Luxilus cornutus*	X	X
Creek chub	*Semotilus atromaculatus*	X	X
Cutlip minnow	*Exoglossum maxillingua*	X	X
Eastern silvery minnow	*Hybognathus regius*	X	X
Emerald shiner	*Notropis atherinoides*	X	X
Fallfish	*Semotilus corporalis*	X	
Fantail darter	*Etheostoma flabellare*	X	X
Fathead minnow	*Pimephales promelas*	X	X
Freshwater drum	*Aplodinotus grunniens*	X	X
Gizzard shad	*Dorosoma cepedianum*	X	X
Golden redhorse	*Moxostoma erythrurum*	X	X
Golden shiner	*Notemigonus crysoleucas*	X	X
Goldfish	*Carassius auratus*	X	X
Grass pickerel	*Esox americanus*	X	
Greater redhorse	*Moxostoma valenciennesi*	X	X
Green sunfish	*Lepomis cyanellus*	X	X
Greenside darter	*Etheostoma blennioides*	X	X
Hornyhead chub	*Nocomis biguttatus*	X	X
Iowa darter	*Etheostoma exile*	X	
Johnny darter	*Etheostoma nigrum*	X	X
Lake sturgeon	*Acipenser fulvescens*	X	X
Lake trout	*Salvelinus namaycush*	X	X
Lake whitefish	*Coregonus clupeaformis*	X	
Largemouth bass	*Micropterus salmoides*	X	X
Logperch	*Percina caprodes*	X	X
Longnose dace	*Rhinichthys cataractae*	X	X
Longnose gar	*Lepisosteus osseus*	X	X
Longnose sucker	*Catostomus catostomus*	X	X
Margined madtom	*Noturus insignis*	X	X
Mimic shiner	*Notropis volucellus*	X	X
Mottled sculpin	*Cottus bairdii*	X	X
Muskellunge	*Esox masquinongy*	X	X
Northern hogsucker	*Hypentelium nigricans*	X	X
Northern pearl dace	*Margariscus nachtriebi*	X	X
Northern pike	*Esox lucius*	X	X
Northern redbelly dace	*Chrosomus eos*	X	
Pumpkinseed	*Lepomis gibbosus*	X	X
Quillback	*Carpiodes cyprinus*	X	X
Rainbow darter	*Etheostoma caeruleum*	X	X
Rainbow smelt	*Osmerus mordax*	X	
Rainbow trout	*Oncorhynchus mykiss*	X	X
Redside dace	*Clinostomus elongatus*	X	X
River Chub	*Nocomis micropogon*	X	
Round goby	*Neogobius melanostomus*	X	X
Rock bass	*Ambloplites rupestris*	X	X
Rosyface shiner	*Notropis rubellus*	X	X
Rudd	*Scardinius erythrophthalmus*	X	
Sand shiner	*Notropis stramineus*	X	X
Shorthead redhorse	*Moxostoma macrolepidotum*	X	X
Silver redhorse	*Moxostoma anisurum*	X	X
Slimy sculpin	*Cottus cognatus*	X	X
Smallmouth bass	*Micropterus dolomieu*	X	X
Splake	*Salvelinus namaycush x Salvelinus fontinalis*	X	
Spotfin shiner	*Cyprinella spiloptera*	X	X
Spottail shiner	*Notropis hudsonius*	X	X
Stonecat	*Noturus flavus*	X	X
Striped shiner	*Luxilus chrysocephalus*	X	X
Tadpole madtom	*Noturus gyrinus*	X	
Tessellated darter	*Etheostoma olmstedi*	X	X
Threespine stickleback	*Gasterosteus aculeatus*	X	X
Tiger muskellunge	*Esox masquinongy x Esox lucius*	X	
Tonguetied minnow	*Exoglossum laurae*	X	X
Trout‐perch	*Percopsis omiscomaycus*	X	X
Walleye	*Sander vitreus*	X	X
Western blacknose dace	*Rhinichthys obtusus*	X	X
White bass	*Morone chrysops*	X	X
White crappie	*Pomoxis annularis*	X	X
White perch	*Morone americana*	X	X
White sucker	*Catostomus commersonii*	X	X
Yellow bullhead	*Ameiurus natalis*	X	X
Yellow perch	*Perca flavescens*	X	X

*Note*: Fish species in the Atlas of Inland Fishes of New York that were determined to be in the Genesee Watershed and in the Genesee River (Carlson et al., 2016). Symbol “X” denotes the presence of the species in the watershed or river that was recorded on a collection map and described in the *Genesee Watershed* subsection for each fish species.
